# The Role of PTEN in Chemoresistance Mediated by the HIF-1α/YY1 Axis in Pediatric Acute Lymphoblastic Leukemia

**DOI:** 10.3390/ijms25147767

**Published:** 2024-07-16

**Authors:** Gabriela Antonio-Andres, Mario Morales-Martinez, Elva Jimenez-Hernandez, Sara Huerta-Yepez

**Affiliations:** 1 Oncology Disease Research Unit, Children’s Hospital of Mexico, Federico Gomez, Mexico City 06720, Mexico; gabya_24@yahoo.com.mx; 2 Department of Medicine, Division of Hematology-Oncology, UCLA David Geffen School of Medicine, Los Angeles, CA 90095, USA; 3 UCLA Jonsson Comprehensive Cancer Center, Los Angeles, CA 90095, USA; 4 Hemato-Oncologia Service, Moctezuma Children’s Hospital, Mexico City 06720, Mexico; elvajimenez@yahoo.com

**Keywords:** PTEN, HIF-1α, YY1, Gp-170, leukemia

## Abstract

Acute lymphoblastic leukemia (ALL) is the most common childhood cancer. Current chemotherapy treatment regimens have improved survival rates to approximately 80%; however, resistance development remains the primary cause of treatment failure, affecting around 20% of cases. Some studies indicate that loss of the phosphatase and tensin homolog (PTEN) leads to deregulation of the phosphoinositide 3-kinase (PI3K)/protein kinase B (Akt) signaling pathway, increasing the expression of proteins involved in chemoresistance. PTEN loss results in deregulation of the nuclear factor kappa-light-chain-enhancer of activated B cells (NF-κB) and induces hypoxia-inducible factor 1-alpha (HIF-1α) expression in various cancers. Additionally, it triggers upregulation of the Yin Yang 1 (YY1) transcription factor, leading to chemoresistance mediated by glycoprotein p-170 (Gp-170). The aim of this study was to investigate the role of the PTEN/NF-κB axis in YY1 regulation via HIF-1α and its involvement in ALL. A PTEN inhibitor was administered in RS4;11 cells, followed by the evaluation of PTEN, NF-κB, HIF-1α, YY1, and Gp-170 expression, along with chemoresistance assessment. PTEN, HIF-1α, and YY1 expression levels were assessed in the peripheral blood mononuclear cells (PBMC) from pediatric ALL patients. The results reveal that the inhibition of PTEN activity significantly increases the expression of pAkt and NF-κB, which is consistent with the increase in the expression of HIF-1α and YY1 in RS4;11 cells. In turn, this inhibition increases the expression of the glycoprotein Gp-170, affecting doxorubicin accumulation in the cells treated with the inhibitor. Samples from pediatric ALL patients exhibit PTEN expression and higher HIF-1α and YY1 expression compared to controls. PTEN/Akt/NF-κB axis plays a critical role in the regulation of YY1 through HIF-1α, and this mechanism contributes to Gp-170-mediated chemoresistance in pediatric ALL.

## 1. Introduction

Acute lymphoblastic leukemia is a malignant neoplasm characterized by the clonal proliferation of hematopoietic precursors within the bone marrow. It represents the most common neoplasm in childhood globally, with recent decades witnessing a survival rate of 90% or higher [1,2]. Despite advancements, treatment failure mechanisms, such as acquired resistance to conventional treatment, known as multi-drug resistance, contribute to therapeutic challenges [3,4]. Resistance mechanisms against drug action often stem from defects within tumor cells, including mutations in genes regulating the cell cycle, such as the tumor suppressor gene *PTEN*, which controls various signaling pathways [5,6,7]. PTEN, or phosphatase and tensin homolog, acts as a tumor suppressor by antagonizing the PI3K-Akt signaling pathway, thereby modulating cell cycle progression and cell survival [7,8]. Somatic alterations in PTEN are prevalent across several human tumors, with its decrease in animal models associated with an elevated tumor formation risk [9,10]. Specific elimination of PTEN in mouse hematopoietic cells leads to the development of acute myeloid leukemia or acute lymphoid leukemia, highlighting its critical role in activating the PI3K/Akt pathway and, consequently, in hematopoietic development and leukemogenesis [11,12]. Moreover, mutations in PTEN, particularly in patients with T-precursor ALL, may correlate with a heightened risk of relapse, potentially linking to resistance mechanisms against conventional treatment schemes, although this remains a subject of debate [13,14,15,16].

The PTEN/PI3K/Akt signaling pathway stimulates the activity of NF-κB, particularly the p65 subunit, enhancing its transcriptional activity. This cascade facilitates the translocation of NF-κB to the nucleus, where it activates target genes associated with antiapoptotic effects, cell growth promotion, and chemoresistance [17,18]. Notably, NF-κB regulates the expression of key proteins such as HIF-1α and YY1 transcription factor, both pivotal in gene expression modulation [19,20,21]. PTEN modulates HIF-1α expression through PTEN/p-Akt signaling, suppressing VEGF expression and angiogenesis across various cancer types [22,23,24]. Additionally, a positive feedback loop exists between PTEN and HIF-1α via miR-21 and the PTEN/Akt/HIF-1α pathway [25,26]. NF-κB can directly regulate YY1 expression by suppressing its gene, with PTEN loss correlating with increased YY1 expression [27,28,29,30,31].

Recent studies from our group revealed HIF-1α’s ability to transcriptionally regulate YY1 expression in pediatric ALL [32]. Moreover, YY1 was found to positively regulate the expression of multi-drug resistance protein Gp-170, strongly linked to chemotherapy resistance in acute lymphoblastic leukemia. Elevated Gp-170 expression, particularly at the nuclear level, is correlated with poorer survival rates [33]. Aberrant activation of YY1 is associated with proliferative and antiapoptotic responses observed in various cancer types. However, the role of the PTEN/NF-κB axis in regulating YY1 via HIF-1α and its involvement in chemoresistance in pediatric ALL remains unclear. This study aims to address these gaps in understanding.

## 2. Results

### 2.1. Inhibition of PTEN Activity Increases HIF-1α and YY1 Expression

**Figure 1:**
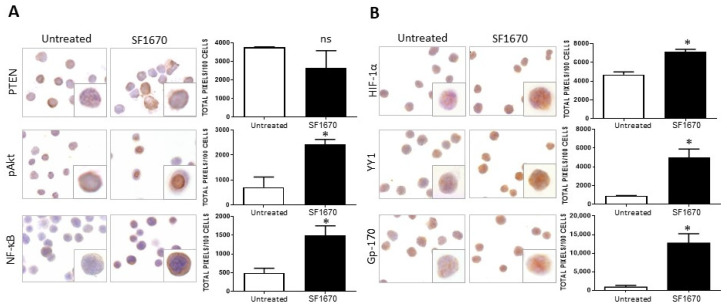
SF1670 treatment decreases PTEN expression in RS4;11 cells. (**A**) Representative microphotograph of PTEN expression in RS4;11 cells treated with the inhibitor SF1670 at 1 µM. A decrease in the expression of PTEN is observed, although it is not statistically significant in RS4;11 cells treated with the inhibitor and an increase in the expression of pAkt, NF-κB, and PTEN (* *p* < 0.05; * *p* < 0.05) compared to untreated cells. (**B**) Expression of HIF-1α, YY1, and gp-170 in RS4 cells; 11 treated with the inhibitor, (HIF-1α * *p* < 0.05; YY1 * *p* < 0.05; Gp-170 * *p* < 0.05; untreated vs. inhibitor). 400× magnification.

The impact of PTEN activity inhibition on HIF-1α, pAkt, and pNF-κB proteins in RS4;11 ALL cells was evaluated. Figure 1A shows a representative photomicrograph of the immunostaining for PTEN in RS4;11 cells treated with the inhibitor SF1670 (1 µM/mL) and cultured for 24 h. When the expression of this protein was quantified, no statistically significant decrease was observed after treatment (Figure 1A). Interestingly, when evaluating the expression of proteins in the signaling cascades that are activated after the inhibition of PTEN activity, such as pAkt and NF-κB, an increase in the expression of these proteins is observed with respect to untreated (Figure 1A). When quantifying the expression, a significant increase was observed for these proteins evaluated both by immunohistochemistry (pAkt * *p* < 0.05, NF-κB * *p* < 0.05). By determining whether the inhibition could infer the expression of proteins that are regulated by NF-κB, such as HIF-1α and YY1, which are downstream of the signaling cascades, it was determined that the use of the inhibitor SF1670 impacts the expression of the aforementioned proteins (Figure 1B), inducing their increase compared to untreated cells (HIF-1α * *p*< 0.05; YY1 * *p* < 0.05). It was determined whether PTEN inhibition had any effect on this glycoprotein Gp-170, which is a drug expulsion pump responsible for conferring chemoresistance to various pharmacological agents. It can be seen that the expression of this protein is modified after treatment with SF1670 (Figure 1B) compared to untreated cells, and we can see that there is an increase compared to the control (* *p* < 0.05).

**Figure 2:**
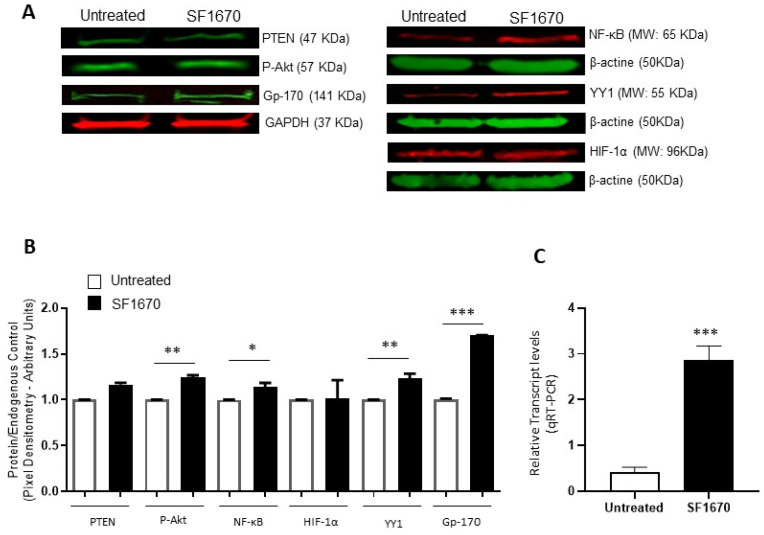
Inhibition of PTEN with SF1670 promotes the expression of NF-κB, YY1, and Gp-170. (**A**) Western Blot representative of the amount of total protein in RS4;11 cells treated with SF1670; the results show differences in the expression of p-Akt and Gp-170 (** *p* < 0.01; *** *p* < 0.001), after treatment with the inhibitor of PTEN. (**B**) The expression of NF-κB and YY1 in the cell line used is modified after treatment with the inhibitor (* *p* < 0.05; ** *p* < 0.01); PTEN does not show changes in the level of protein expression when using the PTEN inhibitor, and HIF-1α has no significant changes. (**C**) Expression of mRNA of YY1 is increased in RS4;11 cells after SF1670 treatment (*** *p* = 0.001 treatment vs. untreated).

To corroborate the above results, protein expression was analyzed using the Western Blot and expression intensity was determined by pixel densitometry (Figure 2). The results show an elevation in the expression of pAkt and Gp-170 (** *p* < 0.01, *** *p* < 0.001, respectively), after treatment with the PTEN inhibitor SF1670 (Figure 2A). NF-κB and YY1 expression show a significantly higher expression elevation due to SF1670 treatment (* *p* < 0.05, ** *p* < 0.01, respectively) (Figure 2B). PTEN shows no change in protein expression level after treatment, and HIF-1α shows a discrete trend for high but not significant protein expression. The levels of mRNA of YY1 also increase after PTEN treatment (Figure 2C).

Taken together, the above results suggest that the PTEN/Akt/NF-κB axis is involved in HIF-1α and YY1 expression and its role in Gp-170-mediated chemoresistance in ALL.

### 2.2. PTEN Inhibition Leads to Sensitization to Chemotherapy Treatment

**Figure 3:**
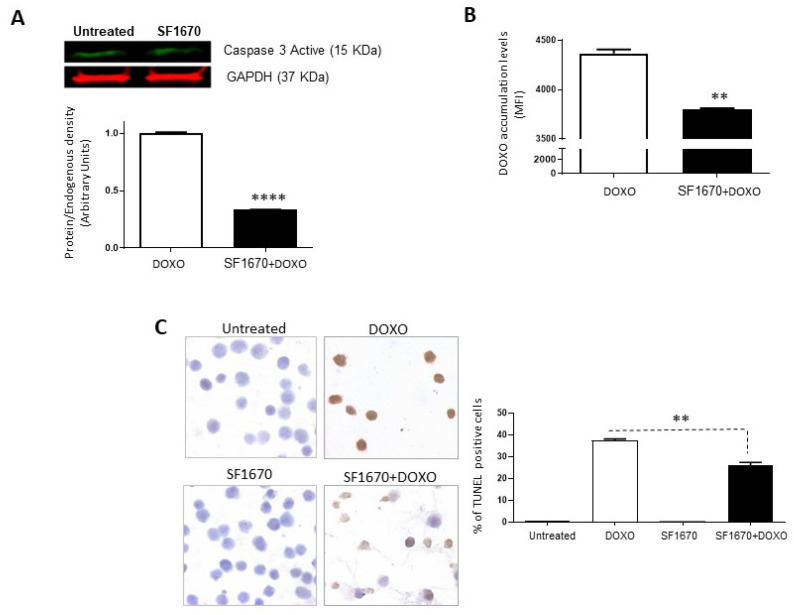
Inhibition of PTEN with SF1670 decreases cell death in RS4;11. (**A**) Western Blot analysis of active caspase-3 from RS4;11 cells treated with SF1670; the results show difference in expression after treatment (**** *p* < 0.0001). (**B**) Doxorubicin accumulation analysis after treatment with the PTEN inhibitor shows a change in the intracellular levels of the drug in RS4;11 cells (** *p* < 0.01). (**C**) Analysis of RS4;11 TUNEL positive cells treated with the inhibitor shows a decrease in TUNEL positive cells in the treatment with PTEN inhibitor and doxorubicin (** *p* < 0.01). 400× magnification.

To determine if the inhibition of PTEN activity by SF1670 leads to a greater sensitivity towards cell death, the presence of active caspase-3 in RS4;11 cells treated with SF1670 was determined (Figure 3A). The results show a difference in the post-treatment expression (*p* < 0.0001). While analysing whether PTEN inhibition can modulate the functionality of Gp-170, the functionality of said glycoprotein was determined by the accumulation of doxorubicin after treatment with SF1670 (Figure 3B); it shows a change in the intracellular levels of the drug in RS4;11 cells (** *p* < 0.01). An analysis was performed on RS4;11 cells treated with SF1670 to determine cell death by TUNEL (Figure 3C); it can be seen that the treatment shows a decrease in TUNEL-positive cells in the treatment with PTEN inhibitor and doxorubicin compared to cells that were treated with doxorubicin (** *p* < 0.01), showing greater sensitivity to cell death and to the action of doxorubicin in cells that do not have PTEN activity inhibited.

### 2.3. Patients with ALL Show Decreased Expression of PTEN and Increased Expression of HIF-1α and YY1

**Figure 4:**
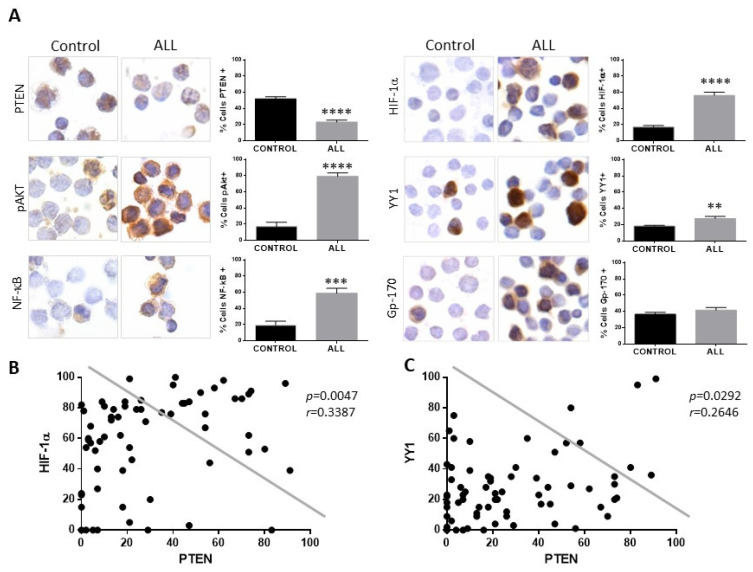
Patients with ALL exhibit decreased expression of PTEN and elevated expression of pAkt, NF-κB, HIF-1α, and YY1. (**A**) PTEN expression in mononuclear cells from pediatric patients with ALL compared with cells from healthy controls showed lower expression of PTEN. An increase in the percentage of cells positive for pAkt, NF-κB, HIF-1, and YY1 was observed in cells from patients with ALL compared to healthy controls (PTEN **** *p* < 0.0001; pAkt **** *p* < 0.0001; NF-κB *** *p* < 0.001; HIF-1α **** *p* < 0.0001; YY1 ** *p* < 0.01; control vs. ALL, respectively) 600× magnification. (**B**) Correlation between the expressions of PTEN and HIF-1α in pediatric ALL, showing a negative correlation between the expression of PTEN and HIF-1α in cells from patients with ALL (Pearson’s test. *r* = 0.3387, *p* = 0.047). (**C**) Correlation between the expressions of PTEN and YY1 in pediatric ALL, showing a negative correlation between the expression of PTEN and YY1 in cells from patients with ALL (Pearson’s test. *r* = 0.2646, *p* = 0.0292).

In the present study, we evaluated the expression of PTEN, HIF-1α, YY1, and Gp-17 proteins in 68 patients with ALL without previous treatment with chemotherapy, as well as 50 healthy controls, and the clinical characteristics of our study population are shown in Supplementary Table S1. Figure 4A shows a representative photomicrograph of the immunocytochemical staining to analyze the expression of PTEN, showing a decrease in the expression of this protein compared to the control group (**** *p* < 0.0001). However, when analysing the expression of proteins involved in the PTEN signaling cascades, it is observed that both pAkt and NF-κB expression in patients with ALL is increased (**** *p* < 0.0001, *** *p*< 0.001). The expression of HIF-1α, YY1 was determined in the samples from patients and controls, and an increase in HIF-1α, YY1, and gp-170 was observed in patients with ALL (Figure 4A) compared to healthy controls. The expression of HIF-1α and YY1 was mainly at the nuclear level, and when quantifying the expression of the proteins, a significant increase in their expression can be observed in patients with ALL compared to healthy controls; **** *p* < 0.0001 for the case of HIF-1α and ** *p* < 0.01 for the case of YY1, although for Gp-170 it is not statistically significant.

**Figure 5:**
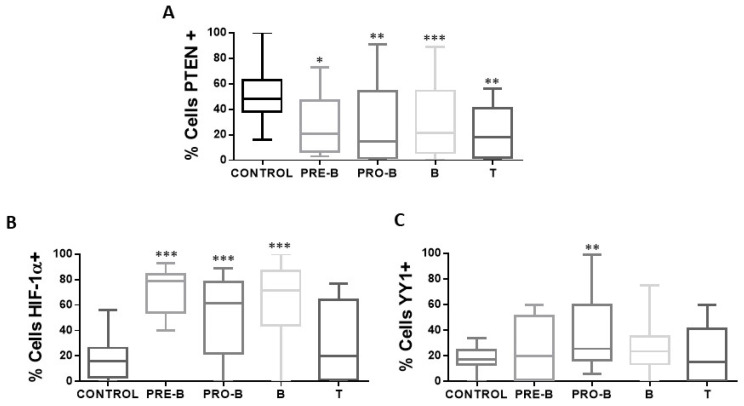
Expression of PTEN, HIF-1α, and YY1 in the cellular immunophenotypes of pediatric ALL. The percentages of positive cells for PTEN, HIF-1α, and YY1 are shown in the different immunophenotypes of patients with ALL (*n* = 68) and control individuals (*n* = 50) in peripheral blood cells of each study subject. (**A**) Shows the expression of PTEN in the different immunophenotypes with a statistically significant increase in the percentage of positive PTEN cells in the samples of patients with ALL with immunophenotype Pro-B, Pre-B, B, and T vs. control (* *p* < 0.05; ** *p* < 0.01; *** *p* < 0.001; ** *p* < 0.01). (**B**) Indicates the expression of HIF-1α in the different immunophenotypes, with a statistically significant increase in the percentage of positive HIF-1α cells in the samples of patients with ALL with Pro-B, Pre-B, and B immunophenotype vs. control (*** *p* < 0.001; *** *p* < 0.001; *** *p* < 0.001). (**C**) Shows the expression of YY1 in the different immunophenotypes, with a statistically significant increase in the percentage of positive YY1 cells in the samples of patients with ALL with immunophenotype Pro-B vs. control (** *p* < 0.01). Comparison was made using unpaired two-tailed *t*-test.

Analysis shows that if there is a correlation between the expression of PTEN with the expression of HIF-1α and YY1 (Figure 4B,C), a negative correlation is also found between the expression of both proteins in relation to the expression of PTEN (PTEN-HIF-1α *p* = 0.0047, PTEN-YY1 *p* = 0.0292). The above results show that there is an increase in the expression of HIF-1α and YY1 in pediatric patients with ALL and a decrease in the expression of PTEN. The expression of PTEN, HIF-1α, and YY1 was also determined between the different phenotypes presented in the patients, and it is shown in the graphs (Figure 5) that there is a decrease in the expression of PTEN in the different phenotypes compared to control individuals (* *p* < 0.05; ** *p* < 0.01; *** *p* < 0.001; ** *p* < 0.01), unlike HIF-1α where there is an increase in the expression of this protein (*** *p* < 0.001; * ** *p* < 0.001; *** *p* < 0.001), except for the T phenotype, while for YY1 only the increase in its expression in the Pro-B phenotype is statistically significant (** *p* < 0.01).

## 3. Discussion

The loss of PTEN function has been implicated in tumor progression events. In this study, inhibiting the activity of this protein in a leukemic cell line affects apoptosis and the efficacy of doxorubicin treatment. Previous biochemical validation of SF1670 (the PTEN inhibitor used in this study) has demonstrated its ability to activate Akt signaling, along with exhibiting a B-cell lineage-specific effect similar to PTEN gene deletion or shRNA-mediated PTEN deletion [34]. This aligns with our findings, as inhibiting PTEN activity with SF1670 did not significantly alter PTEN expression but rather impacted the protein’s function. The effects of PTEN inhibition manifest in the signaling cascades it participates in.

It is well known that the PI3K signaling pathway is crucial for cell growth and survival, activated by membrane receptor tyrosine kinases, leading to PI3K activation and subsequent Akt activation [35]. PTEN regulates this pathway by dephosphorylating PIP3, inhibiting Akt activation [36]. Due to its role in cell proliferation, the PI3K pathway is a target for new anticancer therapies. However, clinical trials show limited efficacy of PI3K inhibitors as monotherapy due to toxicity, poor tolerability, and resistance [35,37,38]. These resistance mechanisms include p85 phosphorylation and PTEN loss, impacting inhibitor effectiveness [38,39,40].

In our study, we observed an increase in Akt activity (pAkt) and NF-κB following PTEN activity inhibition. NF-κB plays a crucial role in tumor proliferation and resistance development, and has been reported to regulate the transcriptional expression of HIF-1α and YY1 transcription factors [27,41]. Additionally, it has been documented that PTEN/PI3K/Akt1 signaling regulates YY1 expression, at least in renal cancer cells [29]. These findings suggest intricate crosstalk between PTEN, Akt, NF-κB, HIF-1α, and YY1 in mediating cellular responses, including apoptosis and chemoresistance, in leukemia.

One of the primary challenges in the success of ALL treatment regimens lies in pharmacological resistance, often associated with increased drug efflux mediated by Gp-170. This phenomenon is linked to gene overexpression and activation of signaling pathways, such as the PI3K/Akt pathway, which is aberrantly active in various cancers, including ALL. This pathway activates NF-κB downstream, largely due to the loss of PTEN functionality [18,42]. Our study demonstrates that inhibiting PTEN functionality results in increased expression of HIF-1α and YY1 proteins, both downstream molecules in the NF-κB activation cascade. This increase correlates with elevated Gp-170 expression, a glycoprotein implicated in expelling cellular cytotoxic agents and contributing to cellular resistance phenotypes [43,44]. This suggests that HIF-1α may enhance this phenomenon, either by directly regulating the promoter of the gene encoding Gp-170 expression or by directly regulating YY1, another transcription factor involved in Gp-170 expression regulation. Our findings indicate that PTEN inhibition enhances Gp-170 activity, as evidenced by the prevention of doxorubicin accumulation [32,33,45]. Our results are consistent with reports indicating that PTEN overexpression is an important event in the regulation of sensitivity to chemotherapy in leukemia cells [18,46].

Recently, many PI3K inhibitors (PI3Kis) have been investigated in clinical trials and approved as potential cancer chemotherapeutic drugs. They are categorized into pan-PI3K inhibitors, isoform-selective PI3K inhibitors, and dual PI3K/mTOR inhibitors [37,47]. For example, the Pan-PI3K inhibitors like pictilisib (GDC-0941) [48], buparlisib (BKM120) [49,50], and copanlisib [51], represent first-generation inhibitors which target all four catalytic isoforms of class I PI3Ks (α, β, γ, and δ). Pictilisib is an oral PI3Ki which has shown efficacy in tumor xenograft models [52] and some clinical trials, common toxicities include nausea, rash, fatigue, and hyperglycemia [50]. Buparlisib is an inhibitor capable of crossing the blood-brain barrier and has an anti-invasive effect in glioblastoma [53]. It has shown some efficacy in hormone receptor-positive/HER2-negative metastatic breast cancer (HR+/HER2− MBC) but is limited by psychiatric and metabolic toxicities [54]. Copanlisib is an intravenous PI3Ki that targets p110α and p110δ isoforms and is FDA-approved for relapsed follicular lymphoma [51] (Commissioner O. DA Approves New Treatment for Adults with Relapsed Follicular Lymphoma. Available online: https://www.fda.gov/news-events/press-announcements/fda-approves-new-treatment-adults-relapsed-follicular-lymphoma, accessed on 14 September 2017) It is being studied in combination with other drugs for advanced HER2+ breast cancer. However, the side effects include hypertension, diarrhea, and transient hyperglycemia [55]. The use of isoform-selective PI3K inhibitors like alpelisib (BYL719), which is an oral isoform-selective PI3Ki targeting the p110α isoform of wild-type PI3Kα yet to be approved by FDA (FDA Approves Alpelisib for Metastatic Breast Cancer. FDA 2019. Available online: https://www.fda.gov/drugs/resources-information-approved-drugs/fda-approves-alpelisib-metastatic-breast-cancer, accessed on 24 May 2019). Compared to the other isoform, alpelisib specificity induces a stronger activity against PI3Kαis and requires a more precise selection of patients but offers improved efficacy and fewer adverse events compared to pan-PI3K inhibitors. This safer profile has led to their development and approval for clinical use [56].

We previously demonstrated that YY1 regulates the transcription of the multi-drug resistance-1 (MDR1/ABCB1) gene, which encodes Gp-170 and plays an important role in chemoresistance [33]. Additionally, we recently showed that HIF-1α transcriptionally regulates, at least in part, the expression of YY1 in ALL cells [32]. PTEN loss results in increased Akt activity (pAkt) and NF-κB activation, leading to the induction of HIF-1α expression in various cancers [57]. This suggests that inhibition of the PI3K signaling pathway, whether with a pan-PI3K inhibitor or an isoform-selective PI3K inhibitor, induces the downregulation of the HIF/YY1/Gp-170 axis and, consequently, reverses chemoresistance. Further studies are needed to confirm this hypothesis.

Regarding patient expression, our findings reveal a decrease in PTEN expression. However, when analyzing its impact on patient survival, we observed no significant change based on this decrease in expression (see Supplementary Table S1). Nonetheless, our previous reports have indicated a significant impact on survival, particularly associated with YY1 expression [58], specifically in pro-B and T immunophenotype ALL.

Some studies have highlighted PTEN’s role in promoting B cell development, homeostasis, and differentiation, which is crucial given that loss of PTEN function can impair recombination and class switch in mature B cells [59]. Oncogenic transformation resulting from loss of PTEN functionality has been extensively studied. Normal B cell development and homeostasis are tightly regulated by PI3K-Akt signaling, although it may have contrasting effects in the pre-B lineage. Targeted PTEN inhibition and hyperactivation of Akt can trigger a checkpoint for autoreactive B cell clearance, offering a potential strategy to overcome drug resistance [34,60]. Our results demonstrate decreased PTEN expression across all B lineage cells, consistent with findings from other authors assessing PTEN expression in B lineage ALL. Similar results were observed in patients with T ALL, aligning with previously published data [13,14], particularly in pediatric patients. Point mutations and loss of PTEN function have been reported in pediatric T ALL patients, leading to reduced PTEN expression due to rapid proteasomal degradation. These anomalies have been associated with poor chemotherapy response, increased relapse risk, and adverse long-term outcomes, although controversies exist regarding the specific anomalies associated with PTEN loss [13,61]. Furthermore, we identified a negative correlation between PTEN expression and the expression of HIF-1α and YY1 in ALL patients. This aligns with findings from PTEN inhibition studies, where decreased PTEN activity led to increased HIF-1α and YY1 expression.

**Figure 6:**
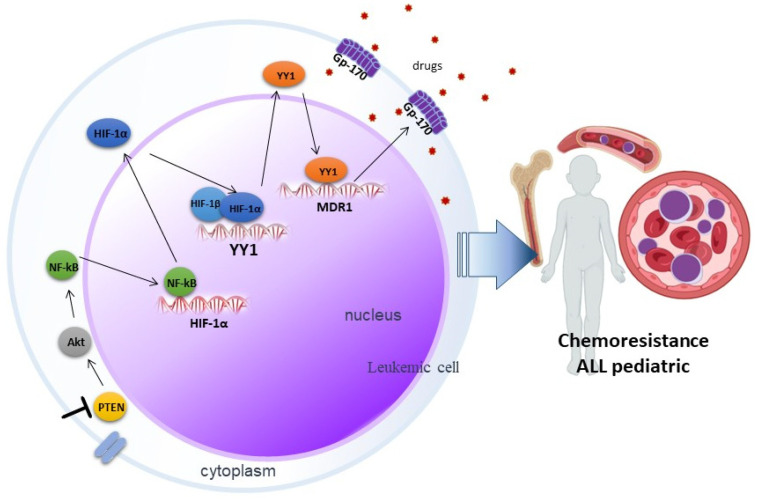
Involvement of PTEN in chemoresistance mediated by the HIF-1α/YY1 axis in pediatric ALL. Inhibition of PTEN through SF1670 promotes the activation of Akt, which leads to the activation of NF-κB, this results in the translocation of transcription factors such as HIF-1α and YY1, which exert their activity on their genes target, importantly in the expression of the glycoprotein Gp-170, which expels drugs and promotes chemoresistance in pediatric ALL.

Much remains to be elucidated in the signaling networks that lead to the development of chemoresistance, but our reported results suggest that inhibition of PTEN via SF1670 promotes Akt activation (Figure 6), leading downstream to activation of NF-κB and, consequently, the modulation in the translocation of transcription factors such as HIF-1α and YY1, which exert their activity on their target genes, leading to the expression of proteins such as Gp-170, which has ejection pump activity that promotes chemoresistance in leukemic cells, which can potentially serve these proteins as therapeutic targets in pediatric ALL.

## 4. Materials and Methods

### 4.1. Study Population

Peripheral blood samples were collected from 68 pediatric patients diagnosed with ALL without prior antineoplastic treatment, aged 0–18 years with a mean age of 8 years, male and female, who were hospitalized at the Hospital Infantil de México Federico Gómez and the Hospital Pediátrico Moctezuma. The samples used were provided by the hemato-oncology service, respectively, while the control samples were obtained from peripheral blood of 50 children who did not present any type of hematological pathology and were provided by the central clinical laboratory service of the Children’s Hospital of Mexico Federico Gómez.

### 4.2. Ethical Considerations

During the development of the project, standards of good laboratory practices were followed to avoid occupational risks due to the handling of patient samples. The study adhered to the regulations of the General Health Research Law of Mexico in force, as well as the research standards of the Hospital Infantil de México Federico Gómez. The Institutional Review Board Statement (research, ethical, and biosafety committees) was approved with the number HIM/2014/018 and under the regulations of the different institutions involved in this project. In each case, an informed consent letter was duly signed. The data provided by the patient’s clinical history were kept confidential, in accordance with the Helsinki international investigation standard.

### 4.3. Sample Processing

Peripheral blood mononuclear cells were purified by means of the gradient separation technique, by centrifugation, using Ficoll-PaqueTM PLUS (BIO-Science AB, Uppsala, Sweden. Cat No. 17-1440-03). Subsequently, slides with 1 × 10^4^ cells were prepared, fixed, and stored for subsequent immunostaining.

### 4.4. Cell Culture and Treatment

The RS4;11 cell line (human acute lymphoblastic leukemia, phenotype B, with growth in suspension; (ATCC: CRL-1873)) was cultured and expanded in a 25 cm^2^ dish with RPMI advanced 1640 medium supplemented with 5% FBS (GIBCO-Invitrogen, Waltham, MA, USA), 1% *v*/*v* L-glutamine (GIBCO-Invitrogen), 1% *v*/*v* Sodium pyruvate (100 mM GIBCO-Invitrogen), 1% antibiotic (GIBCO-Invitrogen), and 1% non-essential amino acids (GIBCO-Invitrogen) and kept in culture at 37 °C and 5% CO_2_.

RS4;11 cells were treated with the PTEN inhibitor SF1670 (C19H17NO3) (Abcam, Cambridge, UK), at a concentration of 1 µM for 24 h at 37 °C. Subsequently, extracts and slides with 1 × 10^4^ cells were collected, prepared, fixed, and stored for subsequent immunostaining.

### 4.5. Immunohistochemistry

Immunostaining was carried out on the slides with cells prepared from patients and from cell cultures. In order to reduce inter-assay variability, all slides were immunostained at the same time. Therefore, antigen retrieval was performed with 0.01 M sodium citrate (pH 6.0), and endogenous peroxidase activity was eliminated with methanol and hydrogen peroxide. It was blocked with normal pig serum (2%) and subsequently, the antibodies, anti-PTEN (Novus Biological, Littletown, CO, USA, AF847), anti-NF-κB (Abcam, Cambridge, UK, ab133112), anti- pAkt (Novus Biological, Littletown, CO, USA, AF887), anti-HIF-1α (Novus Biological, Littletown, CO, USA, NB100-449), anti-YY1 (Novus Biological, Littletown, CO, USA, NBP2-20932), anti-gp-170 (Abcam, Cambridge, UK, ab129450), or anti-Caspase 3 (Abcam, Cambridge, UK, ab32042) in a humid chamber. After washing, it was incubated with a second biotin-conjugated antibody, followed by an incubation with HRP-conjugated streptavidin (Universal LSAB+ KIT/HRP, VECTOR Laboratories, MP-7401). Finally, diaminobenzidine was added and it was counterstained with hematoxylin. After dehydration, the preparations were covered with resin. Slides were analyzed under a microscope (Olympus, BX-40) and positive cells were quantified in 4 fields for each slide (by two investigators) using an image analyzer (Image-Pro Plus^®^, Media Cybernetics, Silver Spring, MD, USA).

### 4.6. Western Blot

Cell lines were grown at a density of 1 × 10^6^ cells/well in culture medium in cell culture plates, lysed with M-PER™ Mammalian Protein Extraction Reagent (Thermo Fischer, Waltham, MA, USA), and cell lysates were quantified with a spectrophotometer using the Lowry method and subsequently denatured at 96 °C, for 10 min. An electrophoretic shift was performed on an acrylamide gel, then transferred to a nitrocellulose membrane using a Trans-Blot turbo (Bio-Rad, Hercules, CA, USA). The membranes were blocked for 1 h under constant agitation, primary antibodies were directed to proteins anti-PTEN (Novus Biological, Littletown, CO, USA, AF847, 1:500), anti-pAkt (Novus Biological, Littletown, CO, USA, AF887, 1:500), anti-Gp-170 (Abcam, Cambridge, UK, ab129450, 1:500), anti-GAPDH (Genetex Alton Pkwy Irvine, CA, USA, GT239), anti-NF-κB (Abcam, Cambridge, UK, ab13311, 1:500), anti-YY1 (Novus Biological, Littletown, CO, USA, NBP2-20932), anti-HIF-1α (Novus Biological, Littletown, CO, USA, NB100-449, 1:500), and anti-B-actin (Genetex Alton Pkwy Irvine, Irvine, CA, USA, GTX110564 1:500). They were incubated overnight under constant agitation at 4 °C and in the dark. Finally, the membrane was washed, shaken with TBS-Tween 20 0.1% (5 min) and incubated with the secondary antibody for one hour and revealed with Oddissey Li-cor equipment. Bands were detected by fluorescence using an Odyssey reader (LI-COR, Lincoln, NE, USA). Image Studio Lit Ver 5.2 software was used to determine protein expression by pixel densitometry. All the raw data from the Western Blots performed are shown in Supplementary Figure S1.

### 4.7. Quantitative Real-Time Polymerase Chain Reaction (qRT-PCR)

For YY1 mRNA expression, RS4;11, 5 × 10^6^ cells were treated or untreated with of the PTEN inhibitor SF1670 (C19H17NO3) (Abcam, Cambridge, UK), at a concentration of 2 µM (RPMI medium) for 24 h at 37 °C (24 Well Cell Culture Plate). After treatment, cells were collected and RNA was purified using the miRNeasy Mini Kit (QIAGEN, Germantown, MD, USA, 217084) according to manufacture instructions. Reverse transcription reactions were conducted with Transcription First Strand cDNA Synthesis Kit (Roche, Indianapolis, IN, USA, 04379012001) using 1 µg of RNA of each sample. The quantitative real-time PCR (qRT-PCR) was performed with SYBR^®^-Green PCR kit (Thermo Scientific, Waltham, MA, USA, 4309155) on an ABI 7500HT real-time PCR system as follows: 95 °C for 5 min; 40 cycles of 30 s of 94 °C and 30 s of 60 °C; and 72 °C for 10 min. The 2^−ΔΔCq^ method was used to analyze the data. Primer sequences were used as follows: YY1 R: 5′-AAAGGGCTTCTCTCCAGT-3′, YY1 F: 5′-TCTCAGATCCCAAACAACT-3′, β-actin R: 5′-GTCCACCGCAAATGCTTCTA-3′, β-actin F: 5′-TGCTGTCACCTTCACCGTTC-3′.

### 4.8. Evaluation of the Functionality of the Gp-170 Pump

To assess the intracellular fluorescence of doxorubicin taken up by RS4;11 cells treated with the PTEN inhibitor SF1670, post-treatment cells were treated for 3 h with 0.25 µM Doxorubicin (Doxolem UK, TEVA). Treated and untreated cells were harvested and fixed with 50 µL Cytofix (BD Pharmingen, Franklin Lakes, NJ, USA). After washing, fluorescence was evaluated using the FACS Calibur flow cytometer. The results were analyzed using FlowJo 8.7 software.

### 4.9. In Situ Detection of Apoptotic Cells (TUNEL)

To assess the effect of PTEN inhibition, doxorubicin was added to cell cultures at a final concentration of 0.25 µM, slides were prepared, and apoptotic cells were identified by the presence of DNA fragmentation with TUNEL staining. Briefly, antigen retrieval was performed with sodium citrate (pH 6, 0.01 M) and endogenous peroxidase activity was blocked with methanol and 3% hydrogen peroxide. It was blocked with 2% normal pig serum, and terminal transferase (Tdt) was diluted in a buffer solution including fluorescein-conjugated oligonucleotides and incubated for 60 min at 37 °C in the dark. Tissues were washed with 1× phosphate buffered saline (PBS) and subsequently incubated with anti-fluorescein antibody at 37 °C for 30 min. Color was developed by adding 3,3’-diaminobenzidine and counterstained with hematoxylin. Samples were dehydrated and mounted on synthetic resin and, once dry, automated digital morphometry analysis was performed.

### 4.10. Statistical Analysis

A database was created, and an exploratory analysis was carried out with all the variables in order to identify the nature of the distribution through a skewness and kurtosis test. Tables and graphs with summary measures, averages, and standard deviations or their non-parametric equivalents were prepared. The degree of association of the response to each protein treatment was estimated by means of a Student’s “*t*” test or a Mann Whitney U test between each group of proteins and an analysis of variance (Bonferroni or equivalents). For non-parametric data, a Mann-Whitney was performed. In addition, a Pearson analysis for correlation was ran. The information was processed using a Prisma 4^®^ statistical analysis program from GraphPad (San Diego, CA, USA).

## 5. Conclusions

Our results collectively demonstrate that the PTEN/Akt/NF-κB axis plays a pivotal role in regulating YY1 via HIF-1α, and this mechanism contributes to Gp-170-mediated chemoresistance in pediatric ALL.

## Data Availability

The original contributions presented in the study are included in the article/Supplementary Materials, further inquiries can be directed to the corresponding author/s.

## Supplementary Materials

The following supporting information can be downloaded at: https://www.mdpi.com/article/10.3390/ijms25147767/s1.
