# Supplementary material for: The Role of PTEN in Chemoresistance Mediated by the HIF-1α/YY1 Axis in Pediatric Acute Lymphoblastic Leukemia
Source: Int J Mol Sci. 2024 Jul 16;25(14):7767. doi: 10.3390/ijms25147767 (PMC11276810; doi:10.3390/ijms25147767)
Supplement: Supplementary file 1 [file ijms-25-07767-s001.zip › ijms-3060845-supplementary.pdf]

A
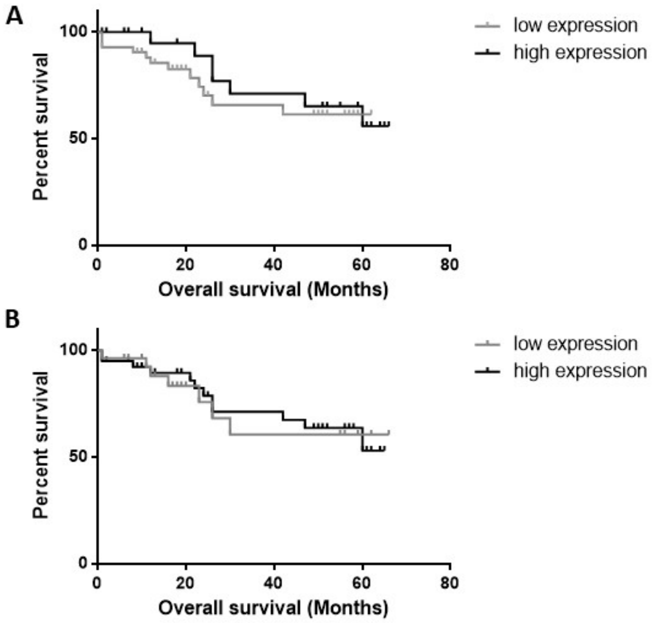

Figure S1. OS according to the positive expression of PTEN (**A**) and HIF-1α (**B**). Univariate survival analysis of high versus low expression intensity effect on OS according to the Kaplan–Meier method.

Supplementary Table S1. Clinical characteristics of Patients.

Characteristics	Value
Total number	68
Gender	
Female	32
Male	36
Age (years)	8.05 (0.1-16)
Phenotype	
B	30
Pre-B	18
Pro-B	11
T	9
Risk	
Standard	14
High	54
Responders	41
With relapse	26
Dead	19
